# Absence of Colony Stimulation Factor-1 Receptor Results in Loss of Microglia, Disrupted Brain Development and Olfactory Deficits

**DOI:** 10.1371/journal.pone.0026317

**Published:** 2011-10-27

**Authors:** Bryna Erblich, Liyin Zhu, Anne M. Etgen, Kostantin Dobrenis, Jeffrey W. Pollard

**Affiliations:** 1 Department of Developmental and Molecular Biology, Albert Einstein College of Medicine, New York, New York, United States of America; 2 Dominick Purpura Department of Neuroscience, Albert Einstein College of Medicine, New York, New York, United States of America; 3 Center for the Study of Reproductive Biology and Women's Health, Albert Einstein College of Medicine, New York, New York, United States of America; Charité Universitaetsmedizin Berlin, Germany

## Abstract

The brain contains numerous mononuclear phagocytes called microglia. These cells express the transmembrane tyrosine kinase receptor for the macrophage growth factor colony stimulating factor-1 (CSF-1R). Using a CSF-1R-GFP reporter mouse strain combined with lineage defining antibody staining we show in the postnatal mouse brain that CSF-1R is expressed only in microglia and not neurons, astrocytes or glial cells. To study CSF-1R function we used mice homozygous for a null mutation in the *Csflr* gene. In these mice microglia are >99% depleted at embryonic day 16 and day 1 post-partum brain. At three weeks of age this microglial depletion continues in most regions of the brain although some contain clusters of rounded microglia. Despite the loss of microglia, embryonic brain development appears normal but during the post-natal period the brain architecture becomes perturbed with enlarged ventricles and regionally compressed parenchyma, phenotypes most prominent in the olfactory bulb and cortex. In the cortex there is increased neuronal density, elevated numbers of astrocytes but reduced numbers of oligodendrocytes. *Csf1r* nulls rarely survive to adulthood and therefore to study the role of CSF-1R in olfaction we used the viable null mutants in the *Csf1* (*Csf1^op^*) gene that encodes one of the two known CSF-1R ligands. Food-finding experiments indicate that olfactory capacity is significantly impaired in the absence of CSF-1. CSF-1R is therefore required for the development of microglia, for a fully functional olfactory system and the maintenance of normal brain structure.

## Introduction

The mammalian brain is populated with a large number of mononuclear phagocytes known as microglia [1], [2]. These cells are found throughout the entire central nervous system at varying densities and with a wide range of morphologies [3]. Microglia/macrophages are found in the neural plate as soon as it is formed [4] but in mice the major increase in microglial number occurs postnatally from post-partum (PP) day 6 [5]. Microglia have been ascribed many roles most frequently relating to immunological, apoptotic cell clearance and repair activities [2], [6], [7], [8]. It has also been suggested that they have a role in neuronal development by guiding axons [9] and eliminating neuronal projections [10]. For example, in neuroendocrine neurons they have been shown to “prune” neurosecretory termini [11]. In addition, roles in neurogenesis [11], [12], [13] and in neuronal survival and synaptogenesis [14], [15] have been proposed.

Macrophages are primarily regulated by the growth factor, colony stimulating factor-1 (CSF-1, also known as macrophage-CSF) [16]. CSF-1 signals via a Class III transmembrane receptor tyrosine kinase (CSF-1R), the product of the *cfms* proto-oncogene [17]. The central role of CSF-1 in macrophage biology was demonstrated in vivo by genetic studies of mice homozygous for the *Csf1* null mutation osteopetrotic (*Csf1^op/op^*) [18] and *Csflr* null mutation [19] as well as toothless (*Tl*) in rats that also lack CSF-1 [20]. In particular evaluation of *Csf1^op/op^* mice showed that the majority of macrophages in the body are lost and many macrophage populations, including those of bone, kidney, testis, and dermis, are almost entirely absent [21]. However, despite the universal expression of the CSF-1 receptor in macrophages, there are some other populations of macrophages that show only small or no alterations in their density through life such as the Langerhans cells in the skin, the resident macrophages in the lung [21], [22] and the microglia in the brain [5], [23]. Thus these macrophages are not reliant on CSF-1 for their development, survival and location. However, even in these cases where macrophages are present in relatively normal numbers their function is compromised in the absence of CSF-1, indicating the need for CSF-1R signaling [24].

Targeted ablation of the *Csf1r* gene in mice also severely depletes macrophage populations. Studies of these mice confirmed that the CSF-1R is the only receptor for CSF-1, as all the phenotypes of the CSF-1 null mutant were found in the receptor null. However, *Csf1r^−/−^* mice have an even more severe phenotype than the *Csf1* null and exhibit poorer viability [19]. The *Csf1r^−/−^* mice have also lost some mononuclear phagocytic populations such as the Langerhans cells that are found in the *Csf1* null [25]. These data suggest another ligand for this receptor. Recently IL-34, was identified and shown to bind to the CSF-1R receptor with high affinity and that can regulate myeloid development and substitute for CSF-1 in vivo [26], [27]. IL-34 has overlapping but not identical biological activities as CSF-1 [28] but shows different spatial and temporal expression patterns [27]. It is likely that this differential expression of IL-34 and CSF-1 can explain the discrepancies in phenotypes between the *Csf1^op/op^* and *Csf1r^−/−^* mice and may explain the differential deletion of some macrophage populations in the CSF-1 ligand and receptor null mutants.

A characteristic of *Csf1^op/op^* and *Csf1r^−/−^* mice is perturbation in development in a wide range of tissues ranging from bone, mammary gland to pancreas [19], [29], [30], [31], suggesting important roles for macrophages in development [32]. In the brain the sex steroid hormone feedback control in the hypothalamus is suppressed and this compromises the hypothalamic-pituitary-gondal (HPG) system and affects reproductive capacity in both sexes [33], [34]. In addition intra-cortical electrophysiological recordings of visual evoked potentials (VEP) that showed that *Csf1^op/op^* mice have primary neuronal abnormalities [14]. These data suggest that CSF-1 plays a role in the development of neuronal circuitry and its absence results in a loss of appropriate connectivity and deficits. However, on a gross histological level *Csf1^op/op^* brains are normal and many behavioral features such as balance and motor functions also appear normal [14]. CSF-1 thus appears to affect specific pathways that result in appropriate neuronal connectivity. As microglia are the primary expresser of CSF-1R in the brain [35], [36], [37], the most parsimonious hypothesis to explain these neuronal defects is that CSF-1 acts through microglia to provide trophic factors to neurons during development. Consistent with this hypothesis, in *Csf1^op/op^* mice fewer microglia are recruited to the sites of injury in the brain and neuronal survival is compromised relative to WT mice receiving comparable injuries [38]. However, CSF-1R expression has also been reported in neurons following ischemic shock, suggesting that these cells might be direct CSF-1 targets at least during injury [38]. The observation that *Csf1r* null mutation causes more severe phenotypes compared to the *Csf1* null also suggests a redundancy with IL-34 as described above. Thus to further study the role of CSF-1R in the brain we employed genetic approaches. For this we used null mutations in the *Csf1r* gene [39], a CSF-1R GFP reporter mouse strain that has been shown to identify all mononuclear phagocytes [40] and the originally described CSF-1 ligand null mutant (*Csf1^op/op^)* [18]. In this report we show that CSF-1R is expressed solely on microglia post-natally and its loss results in a severe depletion of microglia in embryonic to post-weaning mice. CSF-1R is also required for the maintenance of normal brain architecture. In addition we demonstrate that CSF-1 signaling is essential for normal olfaction.

## Results

### Microglia are Strongly Reduced in *Csf1r* Null Mutant Mice

**Figure 1:**
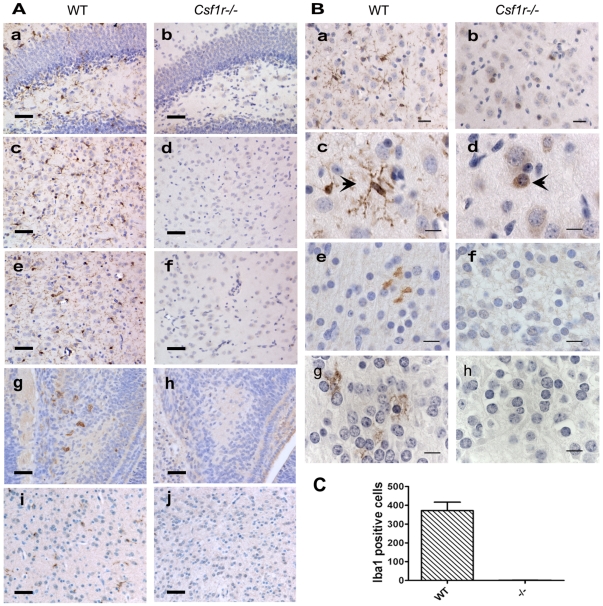
Loss of CSF-1R results in severe depletion of microglia. **A.** Coronal 5 µm sections of brains of 3 week old (a–f), E16 (g, h) and D1 PP (i, j) mice. Wt (a, c, e, g, i) and *Csf1r^−/−^* (b, d, f, h, j) immunostained with anti-Iba1 antibody and counterstained with hematoxylin. Sections from the hippocampus (a, b, g, h), thalamus (c, d, i, j) and preoptic area of the hypothalamus (e,f). Bar 50 µm. **B.** Higher power micrographs of sections of the thalamus (a–d, g, h), Cortex (e,f) from Wt (a, c, e, g,) and *Csf1r^−/−^* mice (b, d, f, h,) immunostained with anti-Ibal (a–f) or anti-F4/80 (g, h) antibodies. Mice at 3 weeks (a–d) and 1 day (e–h) of age. Brown cells in b indicate a cluster of rounded immunostained cells found in *Csf1r^−/−^* mice (b) compared to the highly dendritic cells found in WT mice (a). c and d higher power of individual immunostained cells (arrows) showing the lack of dendritic processes in Iba1 positive cells in *Csf1r^−/−^* mice. a, b (Bar 20 µm), c, d, e, f, g, h (Bar 10 µm). **C.** Enumeration of Iba1 positive cells in a complete half section of the brains of D1 PP WT and *Csf1r^−/−^* (−/−) mice. N = 3/genotype. Significantly different p<0.001.

Microglial numbers plateau within three weeks of birth with all brain regions populated to a varying extent and by cells with differing morphologies [5]. Therefore, we compared mice homozygous for the null mutation in the CSF-1 receptor (*Csf1r^−/−^*) gene to littermate WT mice at 21 PP for the presence of microglia using antibodies against the canonical microglial markers, F4/80 and Iba1. This age was also chosen because the *Csf1r^−/−^* mutants on this genetic background rarely live beyond this age with only occasional mice surviving until 5–6 weeks of age. The mice selected for study were still active and not moribund. Immunostaining with both anti-Iba1 and anti-F4/80 (data not shown) antibodies detected microglia in sections of WT brains (Figure 1A, B). These cells showed characteristic microglial morphology [41] with varying degrees of ramification in all brain areas analyzed (Fig. 1A a,c,e; 1B a,c,) and this pattern was evident with both antibodies. In contrast, microglia in the *Csf1r^−/−^* mice as defined by Iba1 and F4/80 were significantly depleted at 21 PP in all regions analyzed (Fig. 1Ab,d,f, Fig. 1Ba,c).

We performed automated unbiased enumeration of Iba1 expressing cells from different brain regions (Table 1). Iba1 labeled microglia were abundantly found in all regions of the WT mice at varying densities (Table 1). However, consistent with morphological analysis these counts showed that the *Csf1r^−/−^* mice were significantly depleted in Iba1+ cells compared to WT. Indeed in all brain regions of the mutant, most microscopic fields were devoid of microglia indicating severe consequences resulting from CSF-1R loss. However in some regions examined such as the piriform cortex, hippocampus, thalamus and dentate gyrus particularly from two out of the eight mutant mice examined, there were some areas containing clusters of Iba1+ cells (Fig. 1B b,d; Table 1). These bimodial distribution of cell counts are best expressed as medians and this data shows the depletion of Iba1 positive cells in the mutant ranging from 99% in thalamus and hypothalamus to ∼65% in the least affected piriform cortex (Table 1). In each area the difference in Iba1+ cells between WT and *Csf1r^−/−^* was highly significant. Overall when pooled counts from all sections from all areas of the brain were pooled the overall depletion of Iba1+ cells in the brains of *Csf1r^−/−^*mice was ∼94%. Importantly, even the remaining Iba1+ cells in *Csf1r^−/−^* mutants were all phenotypically different from normal WT microglia. Visually (Fig. 1B b,d) and by quantitative morphometry [circularity values of 0.83 (mutant) and 0.73 (WT); p<0.0001; see Methods and Materials) mutant cells were more rounded and relatively lacked the processes seen in the mature normal ramified microglia of WT mice (Fig. 1Ba,c).

**Table 1:** IBA-1 Positive cell numbers in the brains isolated from 3 week old WT and *Csf1r* null mutant mice.

*Region*	*WT*		*Csf1r ^−/−^*		% WT
	# of sections	Median	# of sections	Median	
Caudate nucleus^a^	13	35.5	15	1.8	5.1
Thalamus^a^	11	19.0	15	0.2	1.1
Hypothalamus^b^	10	26.3	15	0.3	1.1
Hippocampus (total)^a^	22	28.8	28	4.0	13.9
Dentate Gyrus^b^	11	29.0	11	5.0	17.2
CA1^c^	11	28.5	17	2.5	8.8
Cortex (total)^a^	21	49	22	7.0	14.3
Piriform Cortex^d^	11	49	11	16.5	33.7
Anterior Cortex^a^	10	46.5	11	6.0	12.9
Total^a^	77	31.5	95	2.0	6.3

Cell Counts of Iba 1 positive cells in the number of sections indicated were made in the brain regions indicated isolated from 7 WT and 8 mutant mice. Median values are presented and percentage of wild type shown.

*Csf1r ^−/−^* statistically different from Wild type.

a p<0.0001;

b p<0.0005;

c p<0.0002:

d p<0.05.

To determine when this depletion occurred we also examined brains for macrophage/monocyte staining at embryonic day (E)16 (Iba1) and 1day PP (F4/80 and Iba1). In WT mice at E16, Iba1 positive cells were detected in the developing brain (Fig. 1A g). However, no labeled cells were detected in the mutant embryonic brains at this stage (Fig. 1Ah). At 1 day PP microglia were evident in all regions of WT mice brains as shown for the thalamus (Fig. 1Ai) and cortex (Fig. 1Be) and was consistent whether F4/80 or Iba1 immunostaining was employed (Fig. 1Ai, Be,g). In contrast, essentially no microglia were found in *Csf1r^−/−^* brains. This was true whether Iba1 or F4/80 antibodies were used and was consistent in six sections from different regions in all three mice examined for each genotype (Fig. 1Aj, 1Bf,h). Quantifying the entire Iba1+ cells under the microscope by manual counting of all antibody-positive cells in one half of the D1 PP brain in coronal sections through the optic chiasma detected less than one positive cell per *Csf1r^−/−^* mouse, highly significantly less than the ∼350 counted in WT mice and the depletion therefore was greater than 99.7% (Fig. 1C).

Iba1 expression has been reported to be independent of microglial activation status [42]. However, CD68 and CD45 are markers for microglial activation and are not expressed by microglia in normal brain [42]. Thus, to determine in the brains of *Csf1r^−/−^* mice whether microglia were activated with aberrant down-regulation of Iba1, we immunostained brains of D1PP mice with anti-CD68 and anti-CD45 antibodies. The positive controls of liver and spleen showed robust immunostaining with both antibodies in a pattern characteristic of Kupffer cells (Fig S1e,f) and splenic macrophages (data not shown) respectively. However, no staining was detected in the brains of either WT mice as expected nor in the brains of *Csf1r^−/−^* (Figure S1a–d). Therefore the loss of CSF-1R does not result in an aberrant activated population of microglia that had lost Iba1 and F4/80 expression. We can conclude therefore through the use of these four markers that the CSF-1R is required for the presence of normal densities and morphology of microglia in the brain from embryogenesis to weaning.

### Disruption of Brain Morphology in *Csf1r* Null Mutant Mice

**Figure 2:**
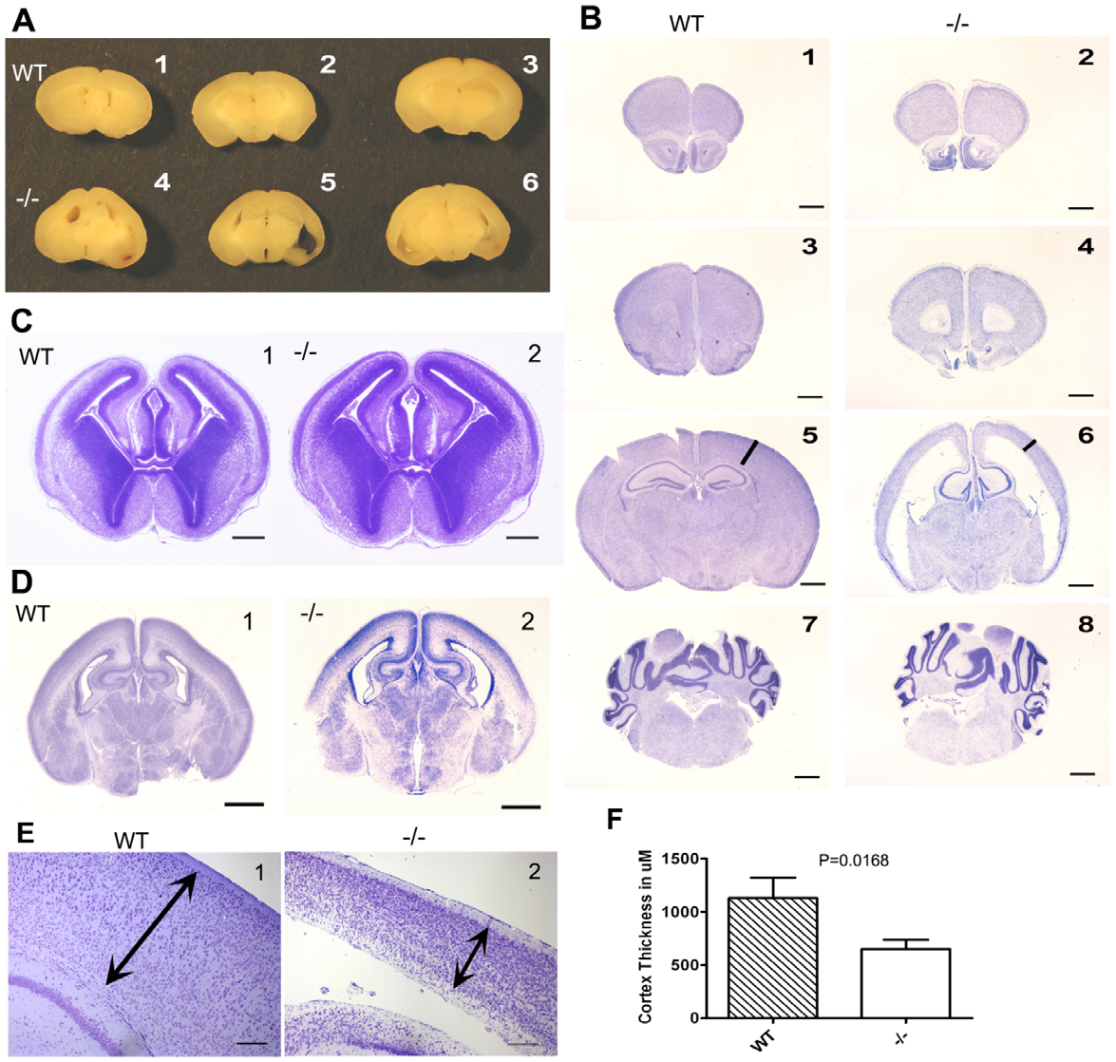
Absence of CSF-1R results in perturbed brain architecture. **A.** Gross architecture of paraformaldehyde fixed brains from 3-week-old mice cut 0.5 mm anterior to the optic chiasm from three WT (1–3) and three *Csf1r^−/−^* (4–6) mice. Note the enlarged ventricles and thinned cortex in *Csf1r^−/−^* mice. **B.** 5 µm Coronal sections stained with Nissl stain. 1, 2, 3, 4, anterior olfactory nucleus. 5, 6, Cortex, thalamus and hippocampus. 7, 8, cerebellum. 1, 3, 5, 7, WT, 2, 4, 6, 8, *Csf1r^−/−^*. Scale bar 1 mm. **C.** 5 µm Coronal sections stained with Nissl stain of brains from WT and *Csf1r^−/−^* E16 mice. Bar = 500 µm. **D.** 5 µm Coronal section through the cortex, thalamus and hippocampus from day 1PP *WT* (1) and *Csf1r^−/−^* (2) mice. Scale bar = 1 mm. **E.** Close up of sections of the cortex from B5 and 6 with lines with double arrows indicating matched regions in which cell counts and measurements were made from WT and *Csf1r^−/−^*. Bar = 200 µm. **F.** Cortical thickness from regions indicated in E. N = 3 for each genotype. p = 0.0168 Student's t test.

**Figure 3:**
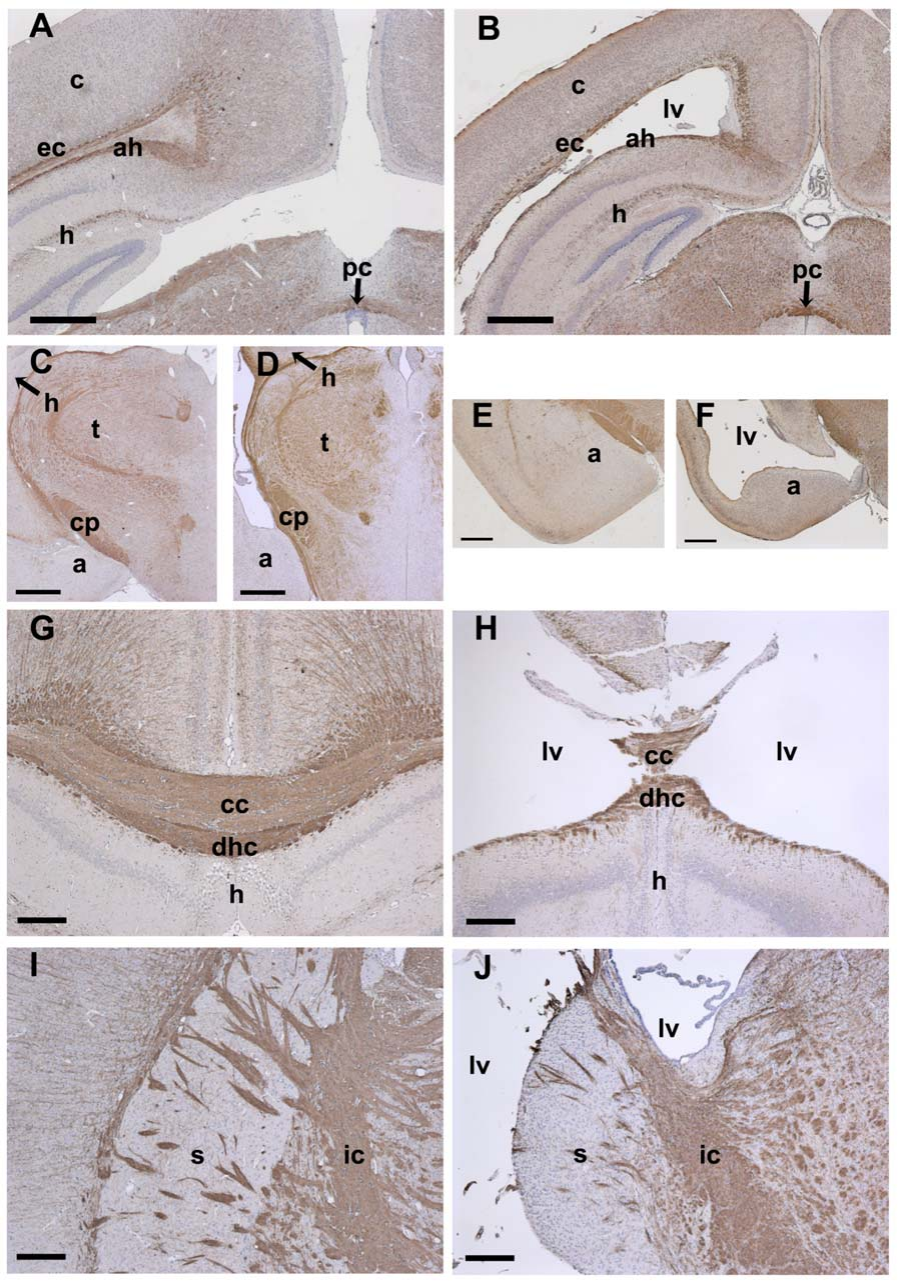
Myelination patterns suggest largely normal late development but with periventricular disruption in *Csf1r^−/−^* mice. Coronal sections from three week old wild type (A,C,E,G,I) and *Csf1r^−/−^* (B,D,F,H,J) mouse brain taken from equivalent co-ordinates along the rostrocaudal axis and immunostained for myelin basic protein (MBP) by immunoperoxidase (brown) and Hematoxylin counterstained (blue). Abbreviations: amygdale (a); alveus hippocampus (ah); cerebral cortex (c); corpus callosum (cc); cerebral peduncle (cp); dorsal hippocampal commissure (dhc); external capsule (ec); hippocampus (h); internal capsule (ic); lateral ventricle (lv); posterior commissure (pc); striatum (s); thalamus (t). (Section in H torn centrally during processing.) Scale bars A–F = 500 µm G–J = 200 µm.

The structural integrity of brains from 3 wk old *Csf1r^−/−^* mice appeared disturbed yielding more fragile specimens. However gross morphological examination showed overall brain size was approximately normal (Fig. 2A) and major brain regions were present as determined using Nissl stained sections (Fig. 2B). However, there was significant ventricular enlargement, particularly notable for the lateral ventricles, with a concomitant reduction in surrounding parenchymal volume (Fig. 2A,B). This reduction was significant in the cerebral cortex, which was thinned circumferentially around the ventricles (Fig. 2B,6) in an apparently proportional manner relative to its normal thickness. In other words, the piriform cortex was thinner compared to the dorsolateral cortex as it is in normal brain. Morphometric measurements of the dorsomedial cortex approximately midway between bregma and interaural zero along the rostro-caudal axis indicated a ∼2-fold reduction in thickness (Fig. 2E,F). The hippocampal region while present, including CA fields, was somewhat reduced in size and appeared distorted both mediolaterally and along the rostrocaudal axis (Fig. 2B5,6 and 3C,D,G,H). Though less striking, careful examination suggested other regions neighboring the lateral ventricles were similarly disturbed including the amygdala and striatum (Fig. 3C–F and 3I,J). The underlying diencenphalon was relatively unaffected (Fig. 2B5,6; 3C,D) suggesting a possible causal relationship between the enlarged lateral ventricular space and aberrations in directly abutting tissue. Enlargement of the cerebrospinal fluid compartment extended into the olfactory ventricle (Fig. 2B1,2; 3,4) along with a volume reduction of the surrounding olfactory area. The olfactory bulb was consistently hollowed out in the *Csf1r^−/−^* mice to the point it often disintegrated during processing. Moving caudally, prominent consistent enlargement of the 3^rd^ or 4^th^ ventricles (Fig. 2B7,8) was not clear nor was an obstruction or occlusion noted that might account for a hydrocephalic-like state. In conjunction, the brain stem and cerebellum did not show significant abnormalities (e.g. Fig. 2B7,8). Relevant to cerebrospinal fluid regulation, choroid plexuses were generally present (e.g. Fig. 2B6,8; 3B,D,J) and Nissl staining showed evidence of a densely packed layer of cells lining the ventricles, consistent with the presence of an ependymal layer (see Figs. 2B and 3).

**Figure 4:**
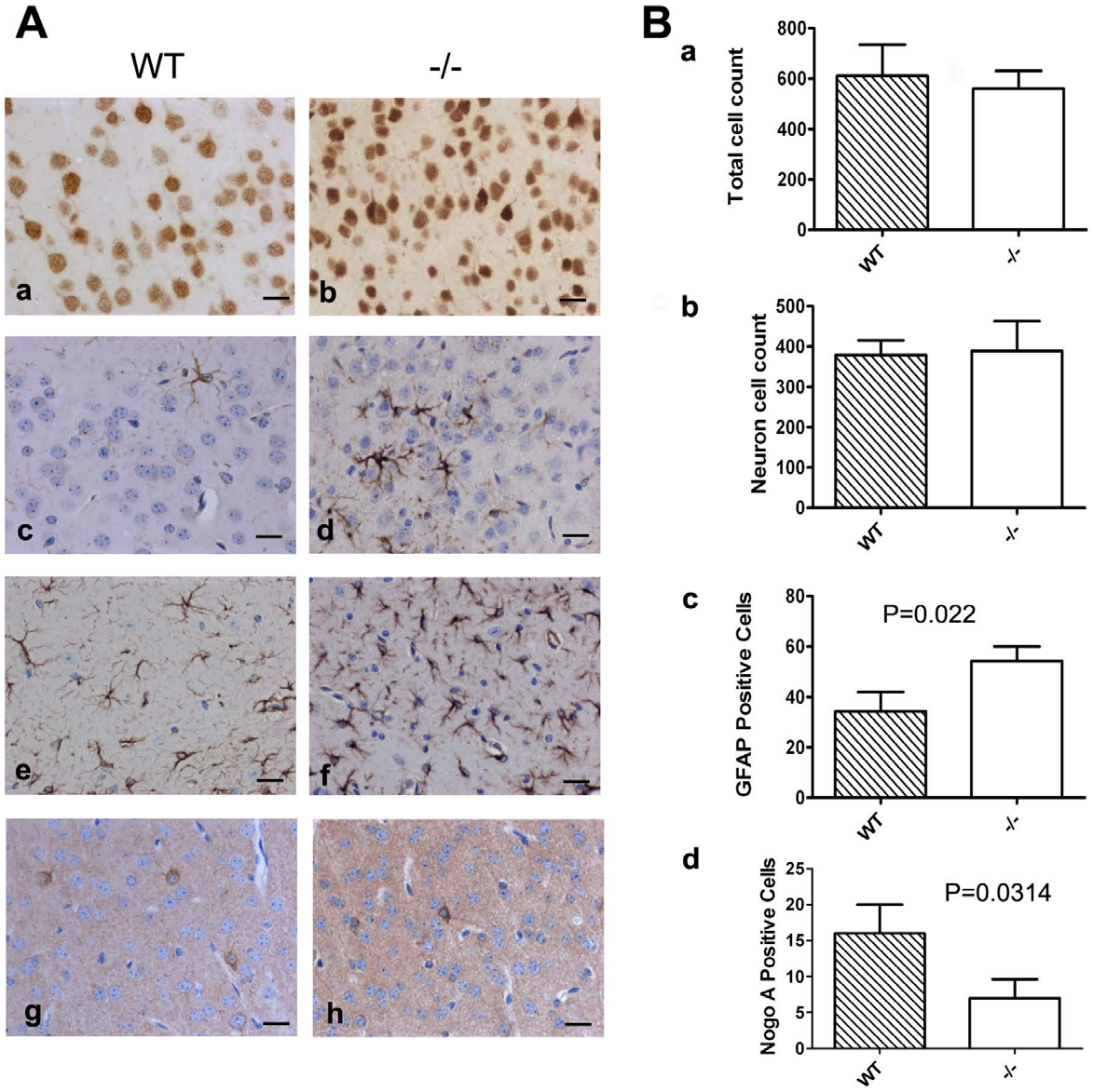
Effects of the loss of the CSF-1R on cell populations in the cortex. **A.** 5 µm sections of cortex (a–d, g,h) and hippocampus (e, f) immunostained with anti NeuN (a, b), anti-GFAP (c–f) or anti-Nogo A antibodies (g,h). c–h counterstained with Haematoxylin. Wild Type (a,c,e,g) and *Csf1r^−/−^* mice (b,d,f,h) Bar = 20 µm. **B.** Total cell counts (N = 3/genotype) determined as described in the materials and methods in the regions indicated in Figure 2B 5, 6 of all Nissl stained cells (a), NeuN positive cells (b), GFAP positive cells (c) and Nogo A positive cells (d). GFAP positive cells are increased in *Csf1r^−/−^* mice compared to WT (p = 0.022) while Nogo A positive ones are decreased (p = 0.0314).

Towards understanding the evolution of the disrupted post-natal state, brain tissue was examined at earlier developmental stages. At E16, the brain appeared relatively normal (Fig. 2C). Neonatal tissue (day 1 PP) grossly showed continued normal development but now included signs of increased lateral ventricular size (Fig. 2D).

To examine in more detail the particularly affected regions, cell type specific immunohistochemical stains as well as cell counts were performed on brains from 3-week old mice. As noted above, the cerebral cortex was notably thinned in *Csf1r^−/−^* mice, but closer examination of Nissl-stained sections suggested a greater density of cells present here in the knockouts (Fig. 2E). Total cell counts were carried out on columnar fields equal in width but spanning the entire depth of matched dorsomedial locations of cerebral cortex in Nissl-stained sections (as shown in Fig. 2E). Enumeration in this way revealed no significant difference in cell number between *Csf1r^−/−^* and WT (Fig. 4Ba). Analogously, NeuN staining showed increased density of neurons in *Csf1r^−/−^* mice (Fig. 4Aa,b) and thus no difference in total number of neurons (Fig. 4Bb). In some thinned out cortical regions (Fig. 2B6, 2E), the enhanced density obscured evaluation of neuronal stratification, but neuronal layers were evident elsewhere (e.g. Fig. 3B), suggesting migration of neuronal precursors had been largely normal.

S100β and GFAP immunostaining to examine glial lineage cells also both revealed an increase in density of positive cells in cerebral cortex and hippocampus (GFAP: Fig. 4Ac–f; similar data not shown for S100β). However, counts of total GFAP-positive cells in cortex showed, unlike that for neurons, significantly more are present in *Csf1r^−/−^* than WT mice (Fig. 4Bc). The intensity of GFAP immunostaining within individual cells also appeared greater in knockout mice, suggesting this might represent a reactive astrogliosis (Fig. 4Ac–f).

Immunostaining for myelin basic protein (MBP) and Nogo A was used to evaluate differentiated myelin and oligodendrocytes, the former being useful in evaluating the degree of relatively later developmental events. As suggested from gross morphologic observations based on Nissl-staining, this myelin staining presented evidence of predominantly normal architectonics and development (Fig. 3). For example, diencephalon and brain stem regions examined were virtually indistinguishable in their myelinated tracts including intact bilateral communicating pathways such as posterior (Fig. 3A,B) and anterior commissures, local cerebral peduncles (Fig. 3C,D) and internal capsule (Fig. 3I,J). The striatum, although somewhat undersized, also showed grossly typical staining patterns and more medially the internal capsule appeared normal (Fig. 3I,J). In line with other observations, structures immediately neighboring the lateral ventricles were the most disturbed and the fragility of these tissues caused tearing making precise comparisons difficult. Nevertheless, for example, the cerebral cortical grey matter showed presence of myelinated axons (Fig. 3A,B), and corpus callosum (Fig. 3G,H) and external capsule (Fig. 3A,B) were evident, but in all cases these were reduced in comparison to WT. Compatible with disturbances in the oligodendrocyte lineage, the total number of identified Nogo1-positive oligodendrocytes in the cerebral cortex was also reduced by ∼50% (Fig. 4Bd). Similarly, the dorsal hippocampal commissure (Fig. 3G,H) and alveus of the hippocampus (Fig. 3A,B) appeared reduced or disintegrating.

### CSF-1 Deficient Mice Have Olfactory Deficits

The highly perturbed olfactory bulb in the *Csf1r^−/−^* mice led us to question the function of CSF-1R in olfaction. However, the lack of viability of the *Csf1r^−/−^* mice precluded such studies in adult mice. We therefore turned our attention to the viable CSF-1 null mice to determine the requirement for CSF-1 signaling in adults. The brains of the CSF-1 ligand null mice are grossly normal and allometrically slightly larger than WT mice [5], [14], [23] with none of the obvious deficiencies described above in *Csf1r^−/−^* mice. Similarly, in contrast to the severe depletion of microglia in *Csf1r^−/−^* mice as reported above, estimates for the number of microglia in *Csf1^op/op^* mice range from no deficiency to approximately 30% depletion [5], [23], [43], [44], [45]. These estimates probably vary according to strain because the severity of the allele depends upon strain background, or they may reflect developmental stage or area analyzed. Nevertheless, in the strain background used in this study there are no differences in microglial number in adult *Csf1^op/op^* mice [5].

**Figure 5:**
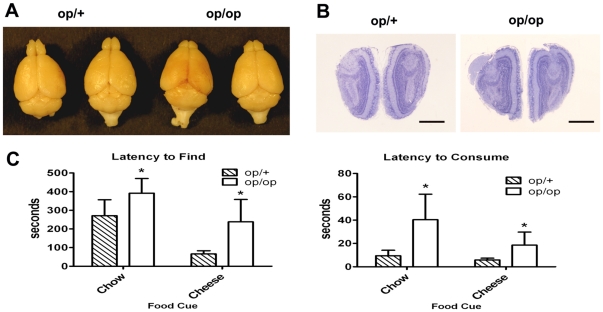
Olfactory Deficits in *Csf1^op/op^* mice. **A.** Representative gross morphology of brains of two WT mice and two *Csf1^op/op^* (*op/op*) mice. Note the runting of the olfactory bulbs in *Csf1^op/op^* mice. **B.** Nissl stained 5 µm sections through the anterior region of olfactory bulbs of WT and *Csf1^op/op^* mice indicate normal morphology in mutant mice. Scale bar = 1 mm. **C.** Olfaction tests: Mice of genotype WT (n = 9) and *Csf1^op/op^* (n = 7) as indicated given a food finding task were challenged on the first night with chow, and on the subsequent evening with cheese. Left panel. Latency in seconds to locate the buried food. Right panel. Latency in seconds to initiate consumption subsequent to finding food. Significant differences between genotypes across food types *p*<0.03 in both panels.

Measurement of the length of the olfactory bulb from rostral to caudal in whole brains that had been carefully removed from adult (12 weeks of age) *Csf1^op/op^* and WT mice indicated that *Csf1^op/op^* bulbs were and ∼66% (p<0.001, n = 3 for each genotype) the length of WT bulbs (Fig. 5A), a phenotype that was missed in earlier studies. This suggests some defect in the development of the olfactory bulbs in the *Csf1* null mice. However, even though the size was truncated there seemed to be no gross morphological differences between WT and mutant mice (Fig. 5B). To measure olfaction we compared *Csf1^op/op^* with WT adult mice in classical olfactory tests involving hidden food challenges. Both WT and mutant mice exhibited similar sniffing behaviors. However, the latency to find buried food was greater in *Csf1^op/op^* versus WT mice (p<0.03; Fig. 5C, left panel), with mutants taking 121 and 172 sec longer to find chow and cheese, respectively. Immediately after finding food, the latency to initiate food consumption was also measured. *Csf1^op/op^* mice took 30.8 and 12.8 sec longer (p<0.03) than WT mice to initiate consumption of chow and cheese, respectively (Fig. 5C, right panel). These experiments indicate that *Csf1^op/op^* mice have olfactory deficits, consistent with a role for CSF-1R signaling in the olfactory system.

### CSF-1R Expressing Cells in Post-Natal Brain are Microglia

**Figure 6:**
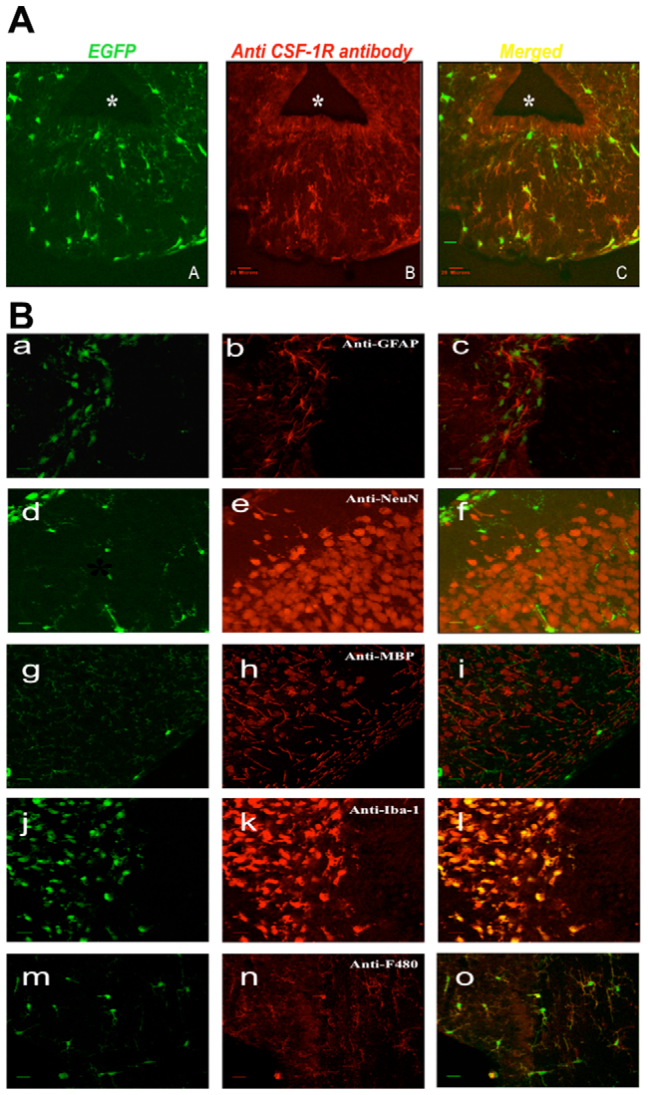
The MacGreen mouse reports CSF-1R expression in the brain and co-localized only with microglia markers. A. Representative 8 µm sections through the hypothalamus of the MacGreen mouse in which GFP reports CSF-1R promoter activity (Panel A) were immunostained with anti-mouse CSF-1R antibody labeled with rhodamine (Panel B). Note GFP is largely cytoplasmic and nuclear while CSF-1R is a membrane protein and therefore labels dendritic processes. Co-localization studies were performed using confocal microscopy and they showed complete co-incidence of labeling (merged image, panel C). * Third Ventricle Bar = 20 µm. B. Representative coronal confocal images of co-localization studies of 8 µm brain sections from the MacGreen mouse in which EGFP is expressed from the *Csf1r* promoter and that marks CSF-1R expressing microglia. Staining for the rhodamine-conjugated antibodies indicated was performed as described in the Materials and methods. Left panels *Csf1r-eGFP* reporter showing GFP labeled cells, Middle panels antibody staining in red, Right panels merged images. Antibody staining does not co-localize with astrocyte (anti-GFAP, a–c), neuronal (anti-NeuN, d–f) or oligodendrocyte (anti-MBP, g–i) markers. In contrast, there is complete congruence with the microglial marker Iba1 (j–l), F480 (m–o). In some cases there appeared to be occasional green cells that were not labeled when viewed on single plane sections in panels g–k. However, upon optical sectioning these were found to co-localize with the immunostain showing that single plane images can be misleading because of differential cellular localization of GFP and the antibody epitope (Data not shown). A-i m,o cortex; j–l hippocampus. Bar = 20 µm.

The data reported above indicate the importance of CSF-1R in the development of microglia, maintenance of the integrity of the brain and for olfaction. However, there has been controversy about which brain cells express the CSF-1R other than microglia and particularly whether neurons are able to express this protein in a functional state [46]. Using the CSF-1R MacGreen reporter mouse in which eGFP is expressed under the control of the *Csf1r* promoter [40], Sierra et al. [37] reported that only microglia express the CSF-1R in adult brain. To confirm and extend these observations we used MacGreen mice to determine whether cells other than microglia express the CSF-1R in the postnatal brain from birth to adulthood. We systematically examined brain regions including cerebellum, cortex, hypothalamus, striatum, and thalamus from at least three independent mice independent *Csf1r-GFP* mice per age at D1 to 17 PP and adulthood (Tables S1 and S2 for details and numbers of mice and antibodies used). The MacGreen mouse marks all mononuclear phagocytes but also, owing to a loss of translational control mechanism, some neutrophils are GFP positive even though these cells don't express the CSF-1R protein [47]. Although neutrophils are excluded from normal brain and are easily recognized by their polymorphonuclear characteristics, we used anti-CSF-1R antibodies [48] to validate that all GFP expressing cells were CSF-1R protein positive in the brain. In all regions tested there was 100% congruence between GFP positive cells and the immunostaining with the anti-mouse CSF-1R antibody (Fig. 6A). Although in thin sections as shown in the Figure 6A there occasionally seem to be cells that do not express the receptor, this is because the GFP is throughout the entire cell with concentration in the nucleus whereas the receptor staining is localized to the membrane. Z series through the labeled cells showed complete congruence in staining (data not shown). The congruence of CSF-1R immunostaining with the GFP expressed from the *Csf1r* promoter (Fig. 6A) was true both during early post-natal development and adult stages (shown for hypothalamus, Fig. 6A). These data confirm that the MacGreen mouse strain accurately and robustly reports CSF-1R expression. Thus MacGreen mice were used to identify CSF-1R expressing cells in the brain.

GFP was not evident in oliogodendrocytes (identified with MBP), astrocytes (anti-GFAP) or neurons (anti-NeuN) in any brain area at any age examined (Fig. 6B, Table 2). Using anti-Iba1 and anti-F4/80 [3], [37] antibodies to define microglia, we found 100% concordance of GFP with both markers in all brain regions and ages tested (Fig. 6B and Table 2). These regions (Table S1 for complete list) included the cerebellum, cortex, hypothalamus, striatum, and thalamus. In some cases occasional green cells appeared unlabeled with microglial markers when viewed in single plane confocal sections. However, acquisition of full Z-series optical planes showed these cells were also in fact co-labeled but missed in single sections due to differential subcellular localization of GFP and the antibody epitope on neighboring planes (data not shown). Thus it can be concluded that the MacGreen mouse only marks microglia, and during all post-natal stages and brain regions the CSF-1R is localized exclusively to microglia.

**Table 2:** *Csf1r*-*GFP* Positive Cells Co-localize with Microglial markers.

Marker	Total cells counted	Total number of CSF-1R+ cells (green)	Total number of antibody labeled cells (red)	Total double labeled cells (yellow)	Percentage of co-localizing cells
**MBP**	120	54	66	0	0%
**GFAP**	131	127	4	0	0%
**NeuN**	504	27	477	0	0%
**F480**	68	68	68	68	100%
**Iba-1**	46	46	46	46	100%

Using Z stacks of immunostained brain sections on the confocal microscope as defined in the material and methods and from regions shown in Table S1, the coincidence of the immunostain with the GFP expressed from the *Csf1r* promoter was determined for the number of cells indicated in column 2.

## Discussion

In this report we show that normal microglia require a functional CSF-1R for their presence in the brain during embryogenesis, at birth and beyond weaning. The loss of CSF-1R also produces perturbations in brain architecture. In addition we show that CSF-1 is required for the development and/or maintenance of normal olfaction in mice.

A major observation in this study is that in the absence of CSF-1R there are essentially no macrophages/microglia (<99% depletion) in the embryonic and early post-natal brain. This was the case in all areas of the brain from embryo until day1 PP post-partum, independent of the markers used. However at three weeks of age while most sections examined and in almost all mice were completely depleted of these cells within a particular brain region there were some sections from two mice that had clusters of positively stained cells. These cells were abnormal, being more rounded than the WT suggesting a compromise in function or viability. However, there was no evidence for activation of the microglia as assessed using CD68 and CD45 markers. This mosaic pattern of microglia in the null mutant may indicate some rescue of the phenotype as development continues as is found in the bone marrow and retina of *Csf1^op/op^* mice [21]. Alternatively there may have been infiltration of Iba1 and F4/80 positive macrophages in response to damage to the brain that was especially evident at three weeks of age. However this hypothesis seems unlikely as macrophages would also be expected to express CD45 while these cells did not. Therefore overall these data suggest that there is some compensatory post-natal development of microglia in the absence of CSF-1R but that the cells are abnormal and unlikely to be fully functional. In the bone of *Csf1^op/op^* mice osteoclasts, another specialized mononuclear phagocyte, are also severely depleted over the post-partum period giving an excluded bone marrow cavity. However, in this tissue post-natal compensation occurs from around four weeks of age with increased numbers of osteoclasts developing and the bone marrow cavity being steadily restored [49]. This compensation is due to the expression of FLT3 ligand that acts through the fms-like tyrosine kinase receptor whose signaling circumvents CSF-1R in the osteoclast [50]. A similar mechanism might be at play in the brain.

Macrophages are detected in the embryonic neural plate from day 7.5 post-coitum and these cells are derived from the yolk sac [51]. This study shows that these embryonic brain macrophages/microglia are dependent on the CSF-1R as none are found at E16. The same dependence on CSF-1R was found in zebrafish where the *panther* mutation in the CSF1R results in a failure of the first wave of yolk sac derived phagocytes to invade the brain [4]. However, in contrast to the zebrafish where subsequent waves of macrophage recruitment are independent of the CSF-1R [4], this study where overall depletion was in the order of 90% at three week of age indicates that the CSF-1R is required at all stages of development for recruitment and expansion of macrophages/microglia into the brains of mice. While this paper has been under review, Merad and co-workers using another *Csf1r^−/−^* strain on the FVB/NJ background also demonstrated the requirement for CSF-1R for the presence of embryonic yolk sac macrophages and microglia in the cortex, hippocampus and striatum during the post-partum period to adulthood. These data together with their lineage tracing experiments, show that yolk sac macrophages populate the brain early in embryogenesis and then throughout life proliferate in situ to give most if not all microglia under the influence of the CSF-1R [52]. These data are consistent with the data reported here and together they indicate the importance of the CSF-1R in microglial biology.

The loss of CSF-1R also results in perturbations in brain architecture. However, early brain development proceeds normally with no gross defects being observed at E16 and modest evidence of an increase in the volume of the ventricular system at D1 PP, particularly the lateral ventricles. Ventricular enlargement becomes more pronounced post-natally such that by 3 weeks of age the lateral ventricles, including their anterior extension into the olfactory bulb, are significantly enlarged and the abutting brain regions are reduced and/or misshapen including the cerebral cortex, olfactory bulb and hippocampus. This late progression of pathology coincides with the time course of greatest expansion of microglial numbers in the normal developing mouse brain [5]. The terminal impact seen could be an outcome of retarded development, a degenerative neurocellular state and/or a true non-atrophic hydrocephalic condition. We favor the latter as the likely predominant contributor given the extent of normal development attained, including organized myelinated tracts, evidence of sustained normal cell numbers in the markedly thinned cerebral cortex suggesting pressure-induced compaction, and that the brain regions most recognizably altered were periventricular. The elevation in numbers and intensity of GFAP-positive astrocytes would also be consistent with a reactive response to such pathology [53], [54], as well as with a degenerative process originating in the parenchyma, but not as readily with interrupted development. It is not clear whether the reduced number of oligodendrocytes found in cerebral cortex are a result of direct physical damage or of changes in this lineage, which fully develops later than neurons and astrocytes, secondary to the accumulating pathology in that region or to reduction of potential trophic influences normally obtained from microglia.

This hydrocephaly may be a communicating form [55] as the ependymal lining appears normal without obvious occlusions or indications of obstructions of the ventricular system. If so, this disturbed fluid regulation may indicate a minor role for microglia and brain macrophages in vascular development [56] including blood-brain barrier differentiation [57]. This disturbed fluid regulation might also result from interference of normal outflow of cerebrospinal fluid by cellular debris, especially high during developmental programmed cell death, that would normally be significantly cleared by phagocytic microglia in the parenchyma [58] and by related macrophages in downstream locations such as perivascular, ventricular, meningeal and arachnoid sites [59], [60], [61].

The exact cause of the death of the *Csf1r* null mice is unknown but may be related to diminishing brain function. These neuroanatomical defects are not overtly present in the *Csf1* ligand null at least on a mixed genetic background, and these mice generally survive for long periods [5], [14], [62]. This strongly suggests that the difference in phenotype is due to IL-34 being the major ligand for the CSF-1R in the brain. This is consistent with the high level of IL34 expression in the brain [27]. Transgenic replacement experiments indicate that IL-34 can compensate for CSF-1 in mice [27]. However, cell culture experiments document differences in signaling outputs from the two ligands [28]. These differences together with the spatial and temporal difference in expression [14], [27], [63] suggest that there will be differences in the requirements for these ligands in the brain. Supporting this contention is that mice lacking CSF-1 display neurological defects manifested by loss of normal sex steroid hormone mediated feedback in the hypothalamus and impaired olfaction as described in this study indicating that IL-34 cannot compensate for these functions [5], [14], [33].

The viability of the *Csf1^op/op^* mice has allowed the biology of CSF-1 to be explored. In these mice previous studies indicated a loss of neuronal function in *Csf1^op/op^* mice confirmed by intra-cortical electrophysiological recordings of visual evoked potentials (VEP) that showed that *Csf1^op/op^* mice have primary neuronal abnormalities characterized by limited responses and dampened recovery compared to wild type (WT) mice [14]. These data suggested both excitatory and inhibitory defects, findings that were confirmed by intra-cortical microinjection of the GABA_A_ antagonist, bicuculline [14]. This study furthered these observations of impaired brain functions by showing that *Csf1^op/op^* mice also have diminished olfaction. Thus CSF-1 signaling is necessary for the establishment of some neuronal circuits during development. In support of this hypothesis is that treatment of *Csf1^op/op^* mice with human recombinant CSF-1 during the first ten days of life significantly reversed the defects associated with hypothalamic functioning [5]. Furthermore, CSF-1 treatment of primary neural cell cultures derived from the cortex, hippocampus, cerebellum and hypothalamus of rats resulted in enhanced neuronal survival and neurite outgrowth. Similar results were obtained in cortical cultures from E l7 mouse brains where process outgrowth was enhanced by addition of CSF-1 in WT but was attenuated in the cultures derived from *Csf1^op/op^* mice [14].

The absence of CSF-1R on neurons but its expression in microglia in post-natal mice shown in this and previous studies [14], [37] is consistent with the hypothesis that CSF-1 responsive microglia play a role in regulating brain development. However, caution needs to be exercised in ascribing all the CSF-1R mutant phenotypes in the brain to loss of microglia. Firstly, microglia are absent in mice null for the transcription factor PU.1 [64] that regulates CSF-1R expression in macrophages [66] without reports of brain abnormalities [65]. However, definitive conclusions on the role of microglia in brain development cannot be drawn from the studies of PU.1 null mice, as their early death in the immediate postpartum period precluded further study of brain morphology. Indeed the observations of relatively normal brain development in PU.1 null mice are consistent with the relatively normal development of the brains in *Csf1r* null mice at least until birth. Secondly, CSF-1R expression in neurons has been reported after brain injury [46]. This neuronal expression of the CSF-1R has recently been confirmed in a model of kainic acid-induced excitotoxic neurodegeneration where CSF-1R was up-regulated in pyramidal neurons and in granule cells of the dentate gyrus. In this model CSF-1 enhanced repair. Furthermore conditional genetic ablation of *Csf1r* in neurons using CaMKIIα-*cre* resulted in enhanced damage after kainic acid injury, confirming its function in neurons (Luo et al., in review). However, our previous studies during normal development found no evidence for CSF-1R mRNA expression by RT-PCR of isolated neurons [14] or CSF-1R immunostaining [5]. Likewise, the current study and that of Sienna et al. [37] using the same reporter mouse strain found no evidence of CSF-1R expression in any cells of the normal brain other than microglia/macrophages in all post-natal stages and brain regions examined. Nevertheless, it is still possible that CSF-1R is expressed in a small sub-population of neurons or progenitors that may not be marked by the MacGreen reporter or in neuronal stem or progenitor cells that are most evident during development and that would not have been observed in this study. Alterations in the fate of progenitor cells and their descendents might explain the altered balance between astrocytes and oligodendrocytes found in the mutant. The relative balance of developmental brain and behavioral defects due to lack of CSF-1 signaling in neurons and/or neural progenitors or microglia will await differential ablation of the *Csf1r* in each cell type during development.

The phenotype of *Csf1^op/op^* null mutants with their perturbed olfaction and disrupted hypothalamic-pituitary-gondal axis is similar to that of human patients with Kallmann's syndrome, a form of hypogonadotropic hypogonadism accompanied by anosmia [67]. The association between reproductive deficits and anosmia reflects the common embryonic origin of olfactory receptor neurons and gonadotropin releasing hormone (GnRH) neurons in the olfactory placode. The axons of the olfactory receptors and the GnRH neurons must migrate through the cribiform plate and into the olfactory bulb; GnRH neurons then continue to migrate along the nervus terminalis to their widely dispersed final destinations throughout the medial preoptic area. Mutations in several different genes (e.g., *KAL1, FGFR1, FGF8, PROK2, PROK2R*) result in failure of olfactory receptor axons and GnRH neurons to transit the cribiform plate, disrupting olfactory bulb morphogenesis and producing hypothalamic infertility [67], [68], [69], reminiscent of *Csf1* and *Csf1r* null mutants. The gross morphological defects of the olfactory lobe in our mutant mice resemble those of mice with a targeted null-mutation of the prokineticin-2 gene (*Prok2*) [68], [70]. Although this might suggest a developmental role for microglia in the migration of GnRH neurons out of the olfactory placode, in contrast with human Kallmann's syndrome patients, adult *Csf1^op/op^* mice have normal GnRH neuron numbers and location within the preoptic area [5], and synthesis of GnRH appears normal (Erblich and Pollard, unpublished). Because prokineticin-2 and its receptors are also implicated in the regulation of circadian rhythms and adult function of GnRH neurons [69], future research might be directed towards elucidating the developmental role of CSF-1 and CSF-1R in establishing the afferent inputs to GnRH neurons that are critical for their normal adult function.

In conclusion, this study demonstrates an essential role for CSF-1R function in the development of microglia and for normal brain architecture. In addition, we have discovered that CSF-1 is essential to the integrity and function of the olfactory system. These findings should open up new avenues for research to better understand the role of CSF-1 in brain biology.

## Materials and Methods

### Mice

Macrophage reporter mice expressing GFP from the *Csf1r* promoter (MacGreen), C57Bl/6N.Gn-*Tg*(*Csf1r-EGFP*)_hume_, CSF-1R deficient, C57Bl/6N.Gn.*Csf1r^tmdex5jwp/tmdex5jwp^* (deleted in exon 5 of the *Csf1r* gene) hereafter referred to as *Csf1r^−/−^* mice and their littermate controls *Csf1r^+/−^ and Csf1r^+/+^*, and CSF-1 deficient C57Bl/6N.Gn. *Csf1^op/op^* mice along with WT *Csf1* ^+*/op*^ littermates (as controls) were bred and genotyped as described [39], [40], [62]. There are no reported heterozygous effects on macrophage number or morphology by the ligand or receptor null [18], [19], [21], [71] and therefore these mice homozygous or heterozygous for the WT allele are referred to as wild type (WT). The Albert Einstein College of Medicine animal use protocol committee approved all experimental procedures that were performed in accordance to the NIH guidelines for the use and care of mice, protocol number 20060205.

### Histology and immunostaining

All mice aged 4 days and older were sedated, perfused intra-cardially with PBS followed by 4% (w/v) paraformaldehyde pH 7.4 made up fresh on the day of the experiment. The mice were decapitated and their brains removed and post-fixed overnight at 4°C in 4% (w/v) paraformaldehyde solution. E 16 and Day 1 PP mice were sedated, immediately decapitated, and brains removed and fixed overnight in the same manner as older mice. Brains were serially sectioned on a vibratome in the coronal plane or paraffin sections made at 5 µm. Sections for histological study were stained either with hematoxylin and eosin or Nissl stain.

For immunofluorescent antibody staining 20 µm floating sections (Table S1 for regions examined, ages and numbers of mice) were incubated with antibodies as described in the text and were detected with fluorescent conjugated secondary antibodies (Table S2 for concentrations and isotypes). Following washes, sections were stained with fluorescent Hoescht (1/10,000 dilution), washed and mounted prior to visualization by confocal microscopy. At each age sections were examined serially from anterior to posterior moving through the entire brain to assess colocalization of antibody markers and GFP. Representative detailed images (≥20 per animal) were taken of each region from the various ages examined. Each representative image was obtained using a 40× objective; 2 µm steps were used to generate a given stack of images. Identification of each region was achieved using the Mouse Brain Atlas [72] as well as by examining patterns of Hoescht staining on individual sections. All data were analyzed using NIH Image-J software. For quantification of co-labeled cells, counts were performed on image stacks captured on the confocal microscope. Cells were counted manually by placing an electronic grid over the images. Cells that intersected the grid were assigned a count, providing a systematic scheme for quantification.

For immunohistochemistry 5 µm coronal sections of paraffin embedded brains were incubated independently with anti-F4/80 [5], anti-ionized calcium-binding adapter molecule 1 (Iba-1), anti-CD68, anti-CD45 (Dako, Carpinteria, CA), anti-GFAP (Santa Cruz, CA), anti-NeuN, anti-MBP (Millipore, Billerica, MA), anti-Nogo A (Santa Cruz, CA) antibodies, washed and specific immunostaining detected with appropriate biotinylated secondary antibody and an avidin-biotin/peroxidase kit (Vector Laboratories, Burlingame, CA) as previously described [5]. In all cases staining using isotype matched antibodies or immunoglobulin from un-immunized animals did not show specific staining (not shown). For Iba1 staining on day 21PP the following regions were analyzed: anterior olfactory nucleus, striatum, anterior cortex/neocortex, preoptic area of the hypothalamus, anterior commissure, thalamus, hippocampus (CA1 and dentate gyrus), hypothalamus, 3rd ventricles, piriform cortex, fimbria, median eminence, cerebellum, brain stem and lateral ventricles. In E16 and day 1PP mice for microglial staining, most of the brain was examined except for the most anterior and posterior regions.

Cell quantification of mutant and WT mice was carried out to compare microglial numbers using Iba-1 antibody staining in paired regions of the brain as defined in Table 1. For brain regions containing layered architectonics (i.e., the cortex and hippocampus) images were taken sequentially in a columnar manner so as to capture all cellular layers. For other regions (e.g., thalamus and hypothalamus) the objective was centered on the given region and 4 images were captured moving around that center point in a clockwise direction. Control sections without primary antibody were photographed and used to establish background thresholds for automated counts. All digital images were taken at 200× magnification with a CCD camera (Magnafire; Optronics) in a color format generated by combining exposures through red, blue and green filters. Using Metamorph image analysis software (Molecular Devices), images were batched by region. They were processed in an automated manner to count Iba-1 positive cell bodies. First, the blue filter component from original RGB color images, taken for display purposes, was isolated and converted to monochrome for analysis. This component was selected as it provided the greatest contrast between DAB staining and unstained tissue, as well as the minimal detection of the blue nuclear counterstaining performed, which could otherwise interfere with Iba-1 counting. Using the “Integrated Morphometry” Metamorph module, Iba-1 positive cells were identified based on pixel intensity values (threshold based on background values from negative control sections), shape factor (roundness), and size. Selected settings were first screened on random images to insure cells were being accurately identified. Thus data from these analyses included the numbers of events (positive cells), as well as descriptive data about the morphometry of the events. Data from the multiple images taken from each section (photographic field = 0.1428 mm^2^) were averaged, yielding a count score for each individual section. Mean and median of counts across the multiple sections in each region were then calculated. Data on the morphology of cells were recorded in a similar manner wherever microglia were present. Because of the non-normal distribution of the count data (many mutant counts were zero), non-parametric analyses (Mann-Whitney U tests) were performed to compare the *Csf1r^−/−^*and WT sections. For cell counts (Nissl, NeuN, Nogo A and GFAP) in the cortex of 3-week old mice all cells were counted through the entire depth with a defined width in the region shown in Figure 2B5 and 6 from at least three different mice of each genotype. At D1 PP the number of anti-Iba 1 stained cells in the entire half of the brain from matched midsections were enumerated from at least three mice of each genotype. Means +/− SD were calculated and compared by Student's t test.

### Assessment of Olfaction

To assess olfactory capabilities, *Csf1^op/op^* and WT littermates were subjected to classic buried food challenges [73], [74], [75]. On the first evening of the tests, each mouse was placed in an arena (86 cm×63 cm×18 cm) for a habituation period. On the second and third evenings, mice were introduced into the test arena where chow and cheese, respectively, were buried and given the opportunity to find food. In the second test, the positioning of the food cues was varied to prevent an animal from locating food based upon memory. Time to find food and the latency to consume was measured on each day. Data for the food-finding olfaction study were analyzed using SAS statistical software package version 9.1.3. A repeated measures factorial ANOVA was employed. Mice with missing values were excluded from analyses.

## Supporting Information

Figure S1 **Microglia in the** ***Csf1^−/−^*** **mice do not express CD45 or CD68.** Representative 5 µm sections of brains from wt (a,c) and *Csf1r^−/−^* (b, d) mice immunostained with anti-CD45 and anti-CD68 antibodies. e, f positive controls of liver showing Kupffer cells immunostained with anti-CD45 (e) and anti-CD68 antibodies (f). Bar = 20 µm.
(TIF)

Table S1 **Brain Regions Examined for Co-Localization of Markers on Microglia.**
(DOC)

Table S2 **Antibodies/Stains Working Concentrations, Sources and Descriptions.**
(DOC)

## References

[1] Hume DA, Perry VH, Gordon S (1983). Immunohistochemical localization of a macrophage-specific antigen in developing mouse retina: phagocytosis of dying neurons and differentiation of microglial cells to form a regular array in the plexiform layers.. J Cell Biol.

[2] Barron KD (1995). The microglial cell. A historical review.. Journal of Neurological Sciences.

[3] Perry VH, Gordon S (1988). Macrophages and microglia in the nervous system.. Trends in Neuroscience.

[4] Herbomel P, Thisse B, Thisse C (2001). Zebrafish early macrophages colonize cephalic mesenchyme and developing brain, retina, and epidermis through a M-CSF receptor-dependent invasive process.. Dev Biol.

[5] Cohen PE, Zhu L, Nishimura K, Pollard JW (2002). Colony-stimulating factor 1 regulation of neuroendocrine pathways that control gonadal function in mice.. Endocrinology.

[6] Glezer I, Simard AR, Rivest S (2007). Neuroprotective role of the innate immune system by microglia.. Neuroscience.

[7] Lalancette-Hebert M, Gowing G, Simard A, Weng YC, Kriz J (2007). Selective ablation of proliferating microglial cells exacerbates ischemic injury in the brain.. J Neurosci.

[8] Streit WJ, Xue QS (2009). Life and death of microglia.. J Neuroimmune Pharmacol.

[9] Milligan CE, Levitt P, Cunningham TJ (1991). Brain macrophages and microglia respond differently to lesions of the developing and adult visual system.. J Comp Neurol.

[10] Berbel P, Innocenti GM (1988). The development of the corpus callosum in cats: a light- and electron-microscopic study.. J Comp Neurol.

[11] Pow DV, Perry VH, Morris JF, Gordon S (1989). Microglia in the neurohypophysis associate with and endocytose terminal portions of neurosecretory neurons.. Neuroscience.

[12] Thored P, Heldmann U, Gomes-Leal W, Gisler R, Darsalia V (2009). Long-term accumulation of microglia with proneurogenic phenotype concomitant with persistent neurogenesis in adult subventricular zone after stroke.. Glia.

[13] Walton NM, Sutter BM, Laywell ED, Levkoff LH, Kearns SM (2006). Microglia instruct subventricular zone neurogenesis.. Glia.

[14] Michaelson MD, Bieri PL, Mehler MF, Xu H, Arezzo JC (1996). CSF-1 deficiency in mice results in abnormal brain development.. Development.

[15] Bessis A, Bechade C, Bernard D, Roumier A (2007). Microglial control of neuronal death and synaptic properties.. Glia.

[16] Chitu V, Stanley ER (2006). Colony-stimulating factor-1 in immunity and inflammation.. Curr Opin Immunol.

[17] Sherr CJ, Rettenmier CW, Sacca R, Roussel MS, Look AT (1985). The c-*fms* proto-oncogene product is related to the receptor for the mononuclear phagocyte growth factor, CSF-1.. Cell.

[18] Wiktor-Jedrzejczak W, Bartocci A, Ferrante AW, Ahmed-Ansari A, Sell KW (1990). Total absence of colony-stimulating factor 1 in the macrophage-deficient osteopetrotic (op/op) mouse [published erratum appears in Proc Natl Acad Sci U S A 1991 Jul 1;88(13):5937].. Proc Natl Acad Sci U S A.

[19] Dai X, Ryan GR, Hapel AJ, Dominguez MG, Russell RG (2002). Targeted disruption of the mouse CSF-1 receptor gene results in osteopetrosis, mononuclear phagocyte deficiency, increased primititive progenitor cell frequencies and reproductive defects.. Blood.

[20] Van Wesenbeeck L, Odgren PR, MacKay CA, D'Angelo M, Safadi FF (2002). The osteopetrotic mutation toothless (tl) is a loss-of-function frameshift mutation in the rat Csf1 gene: Evidence of a crucial role for CSF-1 in osteoclastogenesis and endochondral ossification.. Proc Natl Acad Sci U S A.

[21] Cecchini MG, Dominguez MG, Mocci S, Wetterwald A, Felix R (1994). Role of colony stimulating factor-1 in the establishment and regulation of tissue macrophages during postnatal development of the mouse.. Development.

[22] Qian B, Deng Y, Im JH, Muschel RJ, Zou Y (2009). A distinct macrophage population mediates metastatic breast cancer cell extravasation, establishment and growth.. PLoS ONE.

[23] Blevins G, Fedoroff S (1995). Microglia in colony-stimulating factor 1-deficient op/op mice.. J Neurosci Res.

[24] Guleria I, Pollard JW (2001). Aberrant macrophage and neutrophil population dynamics and impaired Th1 response to Listeria monocytogenes in colony-stimulating factor 1- deficient mice.. Infect Immun.

[25] Merad M, Ginhoux F, Collin M (2008). Origin, homeostasis and function of Langerhans cells and other langerin-expressing dendritic cells.. Nat Rev Immunol.

[26] Lin H, Lee E, Hestir K, Leo C, Huang M (2008). Discovery of a cytokine and its receptor by functional screening of the extracellular proteome.. Science.

[27] Wei S, Nandi S, Chitu V, Yeung YG, Yu W (2010). Functional overlap but differential expression of CSF-1 and IL-34 in their CSF-1 receptor-mediated regulation of myeloid cells.. J Leukoc Biol.

[28] Chihara T, Suzu S, Hassan R, Chutiwitoonchai N, Hiyoshi M (2010). IL-34 and M-CSF share the receptor Fms but are not identical in biological activity and signal activation.. Cell Death Differ.

[29] Wiktor-Jedrzejczak W, Urbanowska E, Aukerman SL, Pollard JW, Stanley ER (1991). Correction by CSF-1 of defects in the osteopetrotic *op/op* mouse suggests local, developmental, and humoral requirements for this growth factor.. ExpHematol.

[30] Felix R, Hofstetter W, Wetterwald A, Cecchini MG, Fleisch H (1994). Role of colony-stimulating factor-1 in bone metabolism.. Journal of Cellular Biochemistry.

[31] Pollard JW, Stanley ER (1996). Pleiotropic roles for CSF-1 in development defined by the mouse mutation osteopetrotic (*op*).. Advances in Developmental Biochemistry.

[32] Pollard JW (2009). Trophic macrophages in development and disease.. Nat Rev Immunol.

[33] Cohen PE, Hardy MP, Pollard JW (1997). Colony-stimulating factor-1 plays a major role in the development of reproductive function in male mice.. Molecular Endocrinology.

[34] Cohen PE, Nishimura K, Zhu L, Pollard JW (1999). Macrophages: important accessory cells for reproductive function.. J Leukoc Biol.

[35] Raivich G, Haas S, Werner A, Klein MA, Kloss C (1998). Regulation of MCSF receptors on microglia in the normal and injured mouse central nervous system: a quantitative immunofluorescence study using confocal laser microscopy.. J Comp Neurol.

[36] Chang Y, Albright S, Lee F (1994). Cytokines in the central nervous system: Expression of macrophage colony stimulating factor and its receptor during development.. Journal of Neuroimmunology.

[37] Sierra A, Gottfried-Blackmore AC, McEwen BS, Bulloch K (2007). Microglia derived from aging mice exhibit an altered inflammatory profile.. Glia.

[38] Fedoroff S, Berezovskaya O, Maysinger D (1997). Role of colony stimulating factor-1 in brain damage caused by ischemia.. Neurosci Biobehav Rev.

[39] Li J, Chen K, Zhu L, Pollard JW (2006). Conditional deletion of the colony stimulating factor-1 receptor (c-fms proto-oncogene) in mice.. Genesis.

[40] Sasmono RT, Oceandy D, Pollard JW, Tong W, Pavli P (2003). A macrophage colony-stimulating factor receptor-green fluorescent protein transgene is expressed throughout the mononuclear phagocyte system of the mouse.. Blood.

[41] Perry VH, Hume DA, Gordon S (1985). Immunohistochemical localization of macrophages and microglia in the adult and developing mouse brain.. Neuroscience.

[42] Luo J, Lin AH, Masliah E, Wyss-Coray T (2006). Bioluminescence imaging of Smad signaling in living mice shows correlation with excitotoxic neurodegeneration.. Proc Natl Acad Sci U S A.

[43] Kondo Y, Lemere CA, Seabrook TJ (2007). Osteopetrotic (op/op) mice have reduced microglia, no Abeta deposition, and no changes in dopaminergic neurons.. J Neuroinflammation.

[44] Sasaki A, Yokoo H, Naito M, Kaizu C, Shultz LD (2000). Effects of macrophage-colony-stimulating factor deficiency on the maturation of microglia and brain macrophages and on their expression of scavenger receptor.. Neuropathology.

[45] Wegiel J, Wisniewski HM, Dziewiatkowski J, Tarnawski M, Kozielski R (1998). Reduced number and altered morphology of microglial cells in colony stimulating factor-1-deficient osteopetrotic *op/op* mice.. Brain Research.

[46] Wang Y, Berezovska O, Fedoroff S (1999). Expression of colony stimulating factor-1 receptor (CSF-1R) by CNS neurons in mice.. J Neurosci Res.

[47] Sasmono RT, Ehrnsperger A, Cronau SL, Ravasi T, Kandane R (2007). Mouse neutrophilic granulocytes express mRNA encoding the macrophage colony-stimulating factor receptor (CSF-1R) as well as many other macrophage-specific transcripts and can transdifferentiate into macrophages in vitro in response to CSF-1.. J Leukoc Biol.

[48] Gouon-Evans V, Rothenberg ME, Pollard JW (2000). Postnatal mammary gland development requires macrophages and eosinophils.. Development.

[49] Begg SK, Radley JM, Pollard JW, Chisholm O, Stanley ER (1993). Delayed hemopoietic development in osteopetrotic (*op/op*) mice.. JExpMed.

[50] Lean JM, Fuller K, Chambers TJ (2001). FLT3 ligand can substitute for macrophage colony-stimulating factor in support of osteoclast differentiation and function.. Blood.

[51] Hume DA (2006). The mononuclear phagocyte system.. Curr Opin Immunol.

[52] Ginhoux F, Greter M, Leboeuf M, Nandi S, See P (2010). Fate mapping analysis reveals that adult microglia derive from primitive macrophages.. Science.

[53] Lee MJ, Chang CP, Lee YH, Wu YC, Tseng HW (2009). Longitudinal evaluation of an N-ethyl-N-nitrosourea-created murine model with normal pressure hydrocephalus.. PLoS One.

[54] Deren KE, Packer M, Forsyth J, Milash B, Abdullah OM (2010). Reactive astrocytosis, microgliosis and inflammation in rats with neonatal hydrocephalus.. Exp Neurol.

[55] Wagner C, Batiz LF, Rodriguez S, Jimenez AJ, Paez P (2003). Cellular mechanisms involved in the stenosis and obliteration of the cerebral aqueduct of hyh mutant mice developing congenital hydrocephalus.. J Neuropathol Exp Neurol.

[56] Checchin D, Sennlaub F, Levavasseur E, Leduc M, Chemtob S (2006). Potential role of microglia in retinal blood vessel formation.. Invest Ophthalmol Vis Sci.

[57] Zenker D, Begley D, Bratzke H, Rubsamen-Waigmann H, von Briesen H (2003). Human blood-derived macrophages enhance barrier function of cultured primary bovine and human brain capillary endothelial cells.. J Physiol.

[58] Napoli I, Neumann H (2009). Microglial clearance function in health and disease.. Neuroscience.

[59] Xiang Z, Burnstock G (2005). Expression of P2X receptors on rat microglial cells during early development.. Glia.

[60] Chinnery HR, Ruitenberg MJ, McMenamin PG (2010). Novel characterization of monocyte-derived cell populations in the meninges and choroid plexus and their rates of replenishment in bone marrow chimeric mice.. J Neuropathol Exp Neurol.

[61] Zhang ET, Richards HK, Kida S, Weller RO (1992). Directional and compartmentalised drainage of interstitial fluid and cerebrospinal fluid from the rat brain.. Acta Neuropathol.

[62] Pollard JW, Hunt JS, Wiktor-Jedrzejczak W, Stanley ER (1991). A pregnancy defect in the osteopetrotic (*op/op*) mouse demonstrates the requirement for CSF-1 in female fertility.. Developmental Biology.

[63] Murase S, Hayashi Y (2002). Neuronal expression of macrophage colony stimulating factor in Purkinje cells and olfactory mitral cells of wild-type and cerebellar-mutant mice.. Histochem J.

[64] McKercher SR, Torbett BE, Anderson KL, Henkel GW, Vestal DJ (1996). Targeted disruption of the PU.1 gene results in multiple hematopoietic abnormalities.. EMBO J.

[65] Beers DR, Henkel JS, Xiao Q, Zhao W, Wang J (2006). Wild-type microglia extend survival in PU.1 knockout mice with familial amyotrophic lateral sclerosis.. Proc Natl Acad Sci U S A.

[66] Hume DA, Wells CA, Ravasi T (2007). Transcriptional regulatory networks in macrophages.. Novartis Found Symp.

[67] Bianco SD, Kaiser UB (2009). The genetic and molecular basis of idiopathic hypogonadotropic hypogonadism.. Nat Rev Endocrinol.

[68] Balasubramanian R, Dwyer A, Seminara SB, Pitteloud N, Kaiser UB (2010). Human GnRH deficiency: a unique disease model to unravel the ontogeny of GnRH neurons.. Neuroendocrinology.

[69] Martin C, Balasubramanian R, Dwyer AA, Au MG, Sidis Y (2010). The Role of the Prokineticin 2 Pathway in Human Reproduction: Evidence from the Study of Human and Murine Gene Mutations.. Endocr Rev.

[70] Cole LW, Sidis Y, Zhang C, Quinton R, Plummer L (2008). Mutations in prokineticin 2 and prokineticin receptor 2 genes in human gonadotrophin-releasing hormone deficiency: molecular genetics and clinical spectrum.. J Clin Endocrinol Metab.

[71] Wiktor-Jedrzejczak W, Ratajczak MZ, Ptasznik A, Sell KW, Ahmed-Ansari A (1992). CSF-1 defeciency in the op/op mouse has differntial effects on macrophage populations and differentiation stages.. Exp Hematol.

[72] Franklin KBJ, Paxinos G (1997). The mouse brain in stereotaxic coordinates.

[73] Baum MJ, Keverne EB (2002). Sex difference in attraction thresholds for volatile odors from male and estrous female mouse urine.. Horm Behav.

[74] Doty RL, Bagla R, Misra R, Mueller E, Kerr KL (2003). No influence of scopolamine hydrobromide on odor detection performance of rats.. Chem Senses.

[75] Yang M, Crawley JN (2009). Simple behavioral assessment of mouse olfaction.. Curr Protoc Neurosci Chapter.

