# Correction: Absence of Colony Stimulation Factor-1 Receptor Results in Loss of Microglia, Disrupted Brain Development and Olfactory Deficits

**DOI:** 10.1371/annotation/5dc1b3a6-ac3c-4c63-951d-cf355ffe7b48

**Published:** 2012-01-27

**Authors:** Bryna Erblich, Liyin Zhu, Anne M. Etgen, Kostantin Dobrenis, Jeffrey W. Pollard

There was an error in the title and citation. The correct title is, "Absence of Colony Stimulating Factor-1 Receptor Results in Loss of Microglia, Disrupted Brain Development and Olfactory Deficits." The correct citation is: Erblich B, Zhu L, Etgen AM, Dobrenis K, Pollard JW (2011) Absence of Colony Stimulating Factor-1 Receptor Results in Loss of Microglia, Disrupted Brain Development and Olfactory Deficits. PLoS ONE 6(10): e26317. doi:10.1371/journal.pone.0026317 

