# Supplementary figures and images for: Absence of Colony Stimulation Factor-1 Receptor Results in Loss of Microglia, Disrupted Brain Development and Olfactory Deficits
Source: PLoS One. 2011 Oct 27;6(10):e26317. doi: 10.1371/journal.pone.0026317 (PMC3203114; doi:10.1371/journal.pone.0026317)

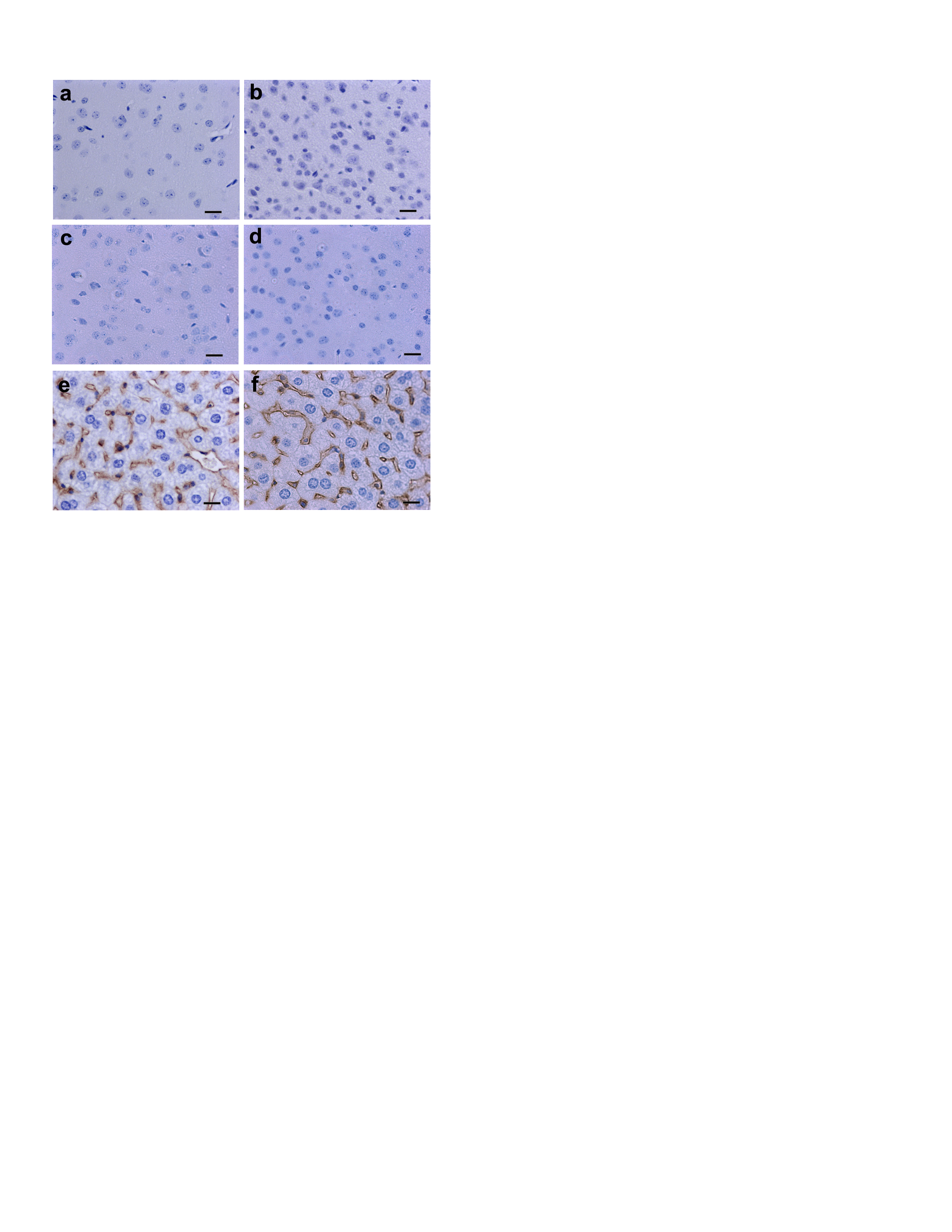

Supplement: Figure S1 — Microglia in the Csf1−/− mice do not express CD45 or CD68. Representative 5 µm sections of brains from wt (a,c) and Csf1r−/− (b, d) mice immunostained with anti-CD45 and anti-CD68 antibodies. e, f positive controls of liver showing Kupffer cells immunostained with anti-CD45 (e) and anti-CD68 antibodies (f). Bar = 20 µm. (TIF) [file pone.0026317.s001.tif]
