# Supplementary material for: Absence of Colony Stimulation Factor-1 Receptor Results in Loss of Microglia, Disrupted Brain Development and Olfactory Deficits
Source: PLoS One. 2011 Oct 27;6(10):e26317. doi: 10.1371/journal.pone.0026317 (PMC3203114; doi:10.1371/journal.pone.0026317)
Supplement: Table S1 — Brain Regions Examined for Co-Localization of Markers on Microglia. (DOC) [file pone.0026317.s002.doc]

**Supplemental Table 1: Brain Regions Examined for Co-Localization of Markers on Microglia**

| **Region** | **Ages Examined** |
| --- | --- |
| Brainstem  Cerebellum  Cortex  Corpus Callosum  Hippocampus  Hypothalamus  -Median eminence  -Preoptic area  Optic Tract  Striatum  Thalamus  Ventricles  -Lateral  -Third | D1, D4, Adult  D4, D7, D10, D14, D17, Adult  D1, D4, D7, D10, D17, Adult  D1, D4, D17, Adult  D1, D4, D7, D17, Adult  D1, D4, D7, D10, D17, Adult  D1, D4, D17, Adult  D1, D4, Adult  D1, D4, D7, D10, D17, Adult  D1, D4, D7, D10, D17, Adult  D1, D4, D7, D10, D17, Adult  D1, D4, D7, D10, D17, Adult  n > 3 mice for each |
