# Supplementary material for: Absence of Colony Stimulation Factor-1 Receptor Results in Loss of Microglia, Disrupted Brain Development and Olfactory Deficits
Source: PLoS One. 2011 Oct 27;6(10):e26317. doi: 10.1371/journal.pone.0026317 (PMC3203114; doi:10.1371/journal.pone.0026317)
Supplement: Table S2 — Antibodies/Stains Working Concentrations, Sources and Descriptions. (DOC) [file pone.0026317.s003.doc]

**Supplemental table 2: Antibodies/Stains Working Concentrations, Source and Descriptions**

| **Antibody/Stain** | **Cell Type Marker** | **Isotype** | **Working Concentration** | **Manufacturer** |
| --- | --- | --- | --- | --- |
| Glial Fibrillary  Acidic Protein  (GFAP) Alexa 568  Neuronal Nuclei  (NeuN) Alexa 488  CD68  Ionizing Calcium  Binding adaptor  Protein 1(Iba-1)  F4/80  Myelin Basic  Protein (MBP)  Nogo A  CSF-1R  Hoechst  Alexa 568  Alexa 594  Alexa 488  Alexa 647 | Astrocyte  Neuron  Activated Microglia  Microglia  Microglia  Oligodendrocyte/  Swann Cells  Oligodendrocyte  Microglia  Nuclei  NA  NA  NA  NA | Mouse  IgG1  Mouse  IgG1  Mouse  IgG1  Rabbit  IgG  Rat IgG  Rabbit  IgG  Rabbit IgG  Rabbit  IgG  NA  Goat anti  Mouse-  IgG1  Goat anti  Rat-IgG  Goat anti  Rabbit-IgG  Goat anti-  Rabbit-IgG | 1/500  1/250 or 1/500  1/25-1/100  1/500  1/50  1/500  1/400  1/250  1:10,000  1:1000  1:250  1:200  1:200 | Molecular probes  Chemicon  Dako  Dako  Self Purified  Chemicon  Santa Cruz  Upstate  Molecular probes  Molecular probes  Molecular probes  Molecular probes  Molecular probes |

NA, not applicable.
